# Siblings of Individuals With Eating Disorders: A Review of the Literature

**DOI:** 10.3389/fpsyt.2020.00604

**Published:** 2020-06-30

**Authors:** Iris Maon, Danny Horesh, Yari Gvion

**Affiliations:** ^1^ Department of Psychology, Bar Ilan University, Ramat Gan, Israel; ^2^ Department of Psychiatry, New York University School of Medicine, New York City, NY, United States

**Keywords:** eating disorders, siblings, brothers, sisters, review

## Abstract

Eating disorders (EDs) are serious psychopathologies characterized by a persistent disturbance in eating or eating-related behavior. Studies have shown EDs’ detrimental consequences not only for patients, but also for their families. Nevertheless, a specific group that has so far been neglected, in both the research and clinical fields, are siblings of individuals with EDs. In an effort to identify this population’s needs, and to facilitate effective prevention and treatment, this paper aims to review the existing literature on the subject, and examine siblings’ personal experience, ways of coping, and levels of psychopathology. PubMed and PsycNet databases were searched with no publication date restrictions, yielding 26 relevant papers. Studies were categorized according to common themes they addressed, and subsequently summarized by highlighting common features, as well as information unique to each study. Several themes emerged, including emotional well-being, psychopathology, social consequences, family dynamics, and coping strategies. Results show that EDs experienced by one individual have significant effects on one’s siblings, such as a decrease in quality of life, social isolation, and elevated familial strain. In several studies siblings were found to have elevated levels of psychopathology and EDs related symptoms. Nevertheless, findings’ nature and magnitude were highly varied. The review indicates the need for further studies that will examine possible intra- and interpersonal moderating factors for EDs’ impact on well-being among siblings, and take into consideration the substantial heterogeneity in studies conducted thus far. Additionally, this review highlights the need for novel and effective interventions, specifically targeting this at-risk group.

## Introduction

Eating disorders (EDs) are a family of psychopathologies characterized by a persistent disturbance of eating or eating-related behavior that results in the altered consumption of food, and significantly impairs physical health or psychosocial functioning (1). The core psychopathology can be conceptualized as an exaggerated, or exclusive, evaluation of self-worth in terms of body shape and weight and the ability to control them, which leads to pathological behaviors such as self-starvation and purging behaviors (2). Three central ED diagnoses are anorexia nervosa (AN), bulimia nervosa (BN), and binge eating disorder (BED), all of which tend to be of persistent nature and have potentially significant and even life-threatening medical consequences (1).

Over the years, studies have shown detrimental consequences of EDs and psychopathology not only for patients themselves, but also for their families (3).

### The Role of the Family in the Etiology and Treatment of EDs

The role of the family in EDs has long been a subject of interest for clinicians and researchers alike. Over the years, family dynamics were studied as a possible factor in the etiology, maintenance, and treatment of EDs. Classic conceptualizations such as Minuchin’s model of the “psychosomatic family” have viewed EDs as an expression of an underlying pathological family structure, characterized by specific interaction patterns such as parental overprotection, rigidity, poor conflict resolution skills, and conflict avoidance (4). These familial processes were seen as playing a causal role in the development of EDs. Indeed, some studies supported the existence of such patterns in families with ED patients (5, 6). Nevertheless, a systematic review of the subject found that although families with ED patients reported worse family functioning than controls, no typical pattern of family dysfunction emerged (7). Furthermore, in a position paper, the Academy for Eating Disorders renounced the use of any etiologic model of EDs in which the primary cause of the disorder is family conduct (5). In spite of the clinical dispute regarding the causal influence of family on EDs, it is widely agreed that involvement of one’s family in the treatment of EDs is recommended, and appears to be useful in reducing psychological and medical symptoms (5).

### Effects of EDs on Family Members

EDs significantly impact not only the individual, but his or her family system as well (3). The intensity and all-encompassing nature of EDs project greatly on family life, influencing members’ personal lives, family relationships and the family’s daily dynamics. Most of the literature on these implications focuses on EDs’ impact on caregiving parents. In a systematic review, parents’ levels of psychological distress, anxiety, and depression, as measured by a variety of instruments, were found to be above clinical cut-off (8).

However, the experiences of siblings in these families, and EDs’ impact on their well-being, seem to be neglected, in both research and the clinical field. We aim to fill this gap in literature by conducting a first-ever review of the literature focusing specifically on siblings of those suffering from EDs. Such examination could aid in identifying unique difficulties in need of more research, and promote the development of preventative interventions and early detection of psychopathology in this group.

### Siblings of Those Coping With Mental Illness

Looking at the field of psychiatry at large, one may find studies examining siblings of individuals with other severe mental illnesses, including schizophrenia and bipolar disorder. Participants reported their siblings’ illness greatly affected their sense of self and their relationships with family members (9). Siblings described a variety of negative emotions such as grief, guilt, fear, and worry (10), leading to significant emotional burden. Accordingly, it has been found that siblings of individuals with mental illness are at elevated risk for emotional, behavioral, social, and developmental impairments (11).

Although psychopathology is commonly regarded as an individual, personal experience by researchers and clinicians alike, studies have consistently shown that symptoms can manifest in those close to the individual, particularly his or her family. The most widely studied psychopathology in this regard is Post Traumatic Stress Disorder (PTSD). Studies have shown those in close proximity to trauma survivors come to develop similar psychological symptoms, without having been directly exposed to the traumatic event (12). Examples of such transmissions were found in children and spouses of Holocaust survivors (13), children of survivors of the Armenian genocide (14), and children and spouses of veterans (15, 16). Similarly, studies found transmission of depression symptoms between parents and their non-genetically-related children (17), and between spouses (18). Finally, transmission of psychotic symptoms and specific delusional beliefs was identified between parents and children, between partners, and between more distant cohabiting family members (19).

Thus, there is empirical evidence for symptom transmission in a variety of psychiatric disorders. However, while the association between mothers’ EDs and EDs among their children has gained some attention (20), its presence in other familial sub-systems such as siblings has been scarcely studied.

As the aforementioned findings suggest, there is reason to assume siblings of individuals with EDs may be at risk for psychopathology in general, and EDs in particular. Similar to other disorders, the continuous stress and burden may harm siblings’ well-being and serve as catalysts for the development of psychopathology (21), alongside other possible pathogenic environmental and genetic factors shared with the siblings coping with EDs (2). Nevertheless, studies concerning siblings’ well-being and psychopathology are scant.

## The Present Review

In most studies involving siblings of individuals with EDs the siblings are referred to solely as a control group, with an intention to understand EDs risk factors and characteristics beyond genetic predisposition (e.g., 22). Studies directly concerning siblings’ experience, characteristics, and well-being have been few and far between. The following paper aims to review both qualitative and quantitative existing literature concerning this group, in order to gain a better understanding of their personal experience, ways of coping, and levels of psychopathology. Better insight into these factors could facilitate more accurate identification and early interventions for those in need of clinical attention.

## Method

### Research Databases and Search Strategy

PubMed and PsycNet databases were searched to identify relevant English-language studies, with no publication date restrictions. The search included a term regarding an ED diagnosis (“eating disorders” or “anorexia” or “bulimia” or “binge eating disorder”), and a term defining the relevant participants (“siblings” or “sisters” or “brothers”), with the Boolean operator “AND” used between the terms. All possible combinations were used. Study titles and abstracts were screened, and if deemed potentially relevant underwent full-text review. In addition, each paper’s reference list was searched for additional relevant papers.

### Study Selection

**Inclusion Criteria:**

(1) Studies including participants with siblings diagnosed with AN, BN, BED, or Eating Disorder Not Otherwise Specified.
(2) Studies that were written in English.
(3) Studies that provided qualitative or quantitative information regarding non-ED siblings. Quantitative studies included a control group other than the diagnosed siblings.

**Exclusion Criteria:**

(1) Studies concerning eating-related disorders which are not psychiatric (e.g., diabetes).
(2) Case reports, commentaries, book chapters, conference papers, and incomplete studies.

### Data Analysis

Studies were first categorized into the following common themes addressed in studies: emotional well-being, psychopathology, eating-related behaviors and symptoms, social consequences, positive consequences, family dynamics, relationship with family members, family role, interaction with health professionals, coping strategies, moderating factors of impact on well-being. Next, we summarized the findings of these studies by highlighting common features in each cluster, as well as information unique to each study.

## Results

The search conducted in electronic databases initially yielded 1,346 papers. After elimination of duplicates, and exclusion of studies that did not meet inclusion criteria, 26 papers were included in the final review (See **Table 1**).

**Table 1:** Core characteristics of reviewed studies.

Study	Ages	Total N	Non-ED siblings’ N	Genders	Exclusion of participants with an ED diagnosis	EDs	Design	Control
Maloney and Shepard-Spiro (23)	17–20	42	21	Male and Female	No past or present AN diagnosis	AN	Cross sectional	Population norms
Casper (24)	17–35	63	15	Female	No past or present AN diagnosis	AN	Cross sectional	Healthy controls
Garley and Johnson (25)	15–18	5	5	Female	No present AN diagnosis	AN	Qualitative	No control group
Stein et al. (26)	N.A	367	42	Male and Female	Not excluded	Various	Cohort study	Healthy controls
Strober et al. (27)	28^a^	905	264	Female	Not excluded	AN, BN	Cohort study	Healthy controls
Latzer et al., (28)	11–18	9	9	Female	N.A	AN	Qualitative	No control group
Wunderlich et al. (29)	12–34	252	84	Male and Female	N.A	Various	Cross sectional	Healthy controls
Benninghoven et al. (30)	21^a^	128	38	Male and Female	N.A	AN, BN	Cross sectional	Healthy controls
Dimitropoulos et al. (31)	25^a^	12	12	Male and Female	N.A	AN	Qualitative	No control group
Areemit et al. (32)	10–18	20	20	Male and Female	No past or present ED diagnosis	N.A	Mixed Methods	No control group
Amianto et al. (33)	26^a^	119	31	Male and Female	No past or present ED diagnosis	AN	Cross sectional	Healthy controls
Rozenstein et al. (34)	23^a^	95	21	Female	No past or present ED diagnosis	AN, BN	Cross sectional	Healthy controls
Rozenstein et al. (35)	23^a^	95	21	Female	No past or present ED diagnosis	AN, BN	Cross sectional	Healthy controls
Dimitropoulos et al. (36)	14–42	52	26	Male and Female	No past or present ED diagnosis	AN	Cross sectional	No control group
Halvorsen et al. (37)	13–35	67	21	Male and Female	No past or present ED diagnosis	AN	Descriptive	Population norms
Degortes et al. (38)	28^a^	267	32	Female	No past or present ED diagnosis	AN	Cross sectional	Healthy controls
Withers et al. (39)	12–18	20	20	Male and Female	N.A	AN	Qualitative	No control group
Latzer et al. (40)	13–31	60	30	Female	No past or present ED diagnosis	Various	Cross sectional	Healthy controls
Steinhausen et al. (41)	N.A	33,185	2,854	N.A	N.A	AN	Cohort study	Healthy controls
Callio and Gustafsson (42)	13–20	5	5	Male and Female	N.A	N.A	Qualitative	No control group
Jungbauer et al. (43)	12–52	16	16	Male and Female	N.A	AN	Qualitative	No control group
Langenberg et al. (44)	16^a^	55	55	Male and Female	N.A	AN	Cross sectional	Population norms
Yao et al. (45)	N.A	2,268,786	N.A	Male and Female	N.A	Various	Cohort study	Healthy controls
Van Langenberg et al. (46)	10–18	12	12	Male and Female	N.A	AN	Qualitative	No control group
Fjermestad et al. (47)	12–23	13	13	Male and Female	N.A	AN	Qualitative	No control group
Weinbach et al. (48)	15^a^	127	20	Female	No present AN diagnosis	AN	Cross sectional	Healthy controls

N.A, Information not available; ^a^Mean age.

### Mental Health and Well-Being Among Siblings of Individuals With EDs

#### Emotional Well-Being

Overall, studies indicated that the ever-present strain and demands of living with a sibling suffering from an ED may have an effect on one’s emotional well-being. In a study examining non-ED siblings’ quality of life, while most subjects did not reach an “at risk” score on the quality of life measurement, 80% of siblings did report a decrease in quality of life brought about by the disorder (32). Non-ED siblings also described the physical effect the disorder has had on them, feeling sick more often, experiencing sleeping problems, having lower energy levels, and experiencing difficulties with attention and concentration (31, 42, 44). Siblings also reported a decline in scholastic functioning, and a decrease in motivation for social activities (28). Additionally, siblings of individuals with EDs described various negative emotions. Feelings of fear and worry about the future were a recurring theme throughout many qualitative studies (25, 28, 31, 32, 43, 47). In one study, 81% of siblings reported they were afraid their sister suffering from AN will never get better, and 43% were afraid she will die (37). Interviews indicated that the diagnosed siblings’ negative behaviors, the inability to help and make them recognize their own disorders, and the feeling that they do not wish to get better have led to feelings of helplessness, sadness, and anger (25, 31, 42, 43, 47). Guilt was also a prominent emotion among non-ED siblings. Some felt responsible for the onset of the ED due to their past remarks or actions, some blamed themselves for not helping their siblings as much as they could, and for some the split loyalty, to their patents on one hand and to their siblings on the other, evoked a persistent sense of guilt (25, 31, 32, 43, 46). Moreover, in-depth interviews with non-ED siblings revealed a sense of grief and sacrifice, as well as a feeling they lost their family, their normal childhood, their good relationship with their sibling, and their individual identity (32, 42, 43).

#### Psychopathology

Overall, findings regarding siblings’ psychopathology have been inconsistent. Some quantitative studies indicated non-ED siblings did not differ from controls on levels of psychological distress, depression, obsessive symptoms, or problems with peers (24, 34, 35, 38, 40, 44). One study even found parents’ reports of non-ED siblings’ conduct problems were lower than population norms (44). However, other studies have found siblings’ self-report of emotional difficulties, obsessive-compulsiveness, and depression was higher than controls’ (33, 40, 44). Studies of familial aggregation of psychopathology showed siblings of ED patients were 1.4 more likely to attempt suicide in comparison to controls (45). They were also significantly more likely to be diagnosed with affective disorders (OR = 1.69), anxiety disorders (OR = 1.54), obsessive-compulsive disorder (OR = 2.04), and personality disorders (OR = 1.62) (41).

#### Eating-Related Behaviors and Symptoms

Findings pertaining to the effects of EDs on the non-ED siblings’ eating behaviors and EDs symptoms were also inconsistent. Some studies showed non-ED siblings did not face an increased risk for the development of EDs in comparison to controls. A family aggregation study found that siblings of AN or BED probands were not more likely than controls to be diagnosed with an ED (26). Other quantitative studies have found that non-ED siblings had no pathological ED symptoms, were similar to controls in their attitudes towards food and eating behaviors, and did not differ from controls in their body image (23, 29, 30, 33, 34, 48). Several qualitative studies supported these findings, as some non-ED siblings denied influence of the siblings’ ED on these psychological aspects, and even claimed to have developed a more positive relationship with their bodies and with food (32, 42). In some interviews, siblings also mentioned holding negative attitudes towards dieting, and becoming more aware of the importance of healthy eating and living (28, 42, 47). Nevertheless, other studies indicated a higher inclination to sub-clinical levels of ED symptoms in non-ED siblings, with some studies reporting that 9.5–12% of siblings scored within the clinical range of EDs (23, 36). In addition, there were studies in which non-ED siblings were found to have a higher level of dissatisfaction with their body as compared to controls (24), and experienced a negative impact of the disorder on their body image (43). In some qualitative studies non-ED siblings reported having issues with eating themselves, becoming more self-conscious of their eating habits, experimenting with different eating behaviors, and holding themselves to their siblings’ standards regarding eating habits and body (25, 32, 37, 43, 47). Using computer simulation for perceived and desired body images, one study found that male siblings of ED patients perceived themselves as having more fat than muscle in comparison to controls (30). Cohort studies also identified an increased risk for EDs among siblings of ED patients. Forty three percent (43%) of sisters of BN patients endorsed a lifetime diagnosis of some ED, and a lifetime diagnosis of AN was four times more common in siblings of patients with AN compared to controls (26, 41). Partial AN and BN were diagnosed in 3.6 and 4.0% of siblings of persons with these diagnoses, respectively (27).

#### Social Consequences

Overall, studies indicated that having a sibling with an ED affected non-ED siblings’ inter-personal relations and social life. While in some studies siblings reported turning to their friends for help and comfort (25, 31, 42), others found siblings avoided disclosing their feelings and experiences outside the family (39). This secrecy sometimes stemmed from parents’ wish to conceal the diagnosed sibling’s status (32), or alternatively from the non-ED sibling’s own sense of embarrassment and shame, or from fear of stigma and of being perceived as weak (32, 39, 42). As was found in several qualitative studies, non-ED siblings felt socially isolated at times. They described a tendency to withdraw from social contact and seldom invited friends over, in an effort to avoid questions and comments about their siblings (25, 42, 47).

#### Positive Implications of Having a Sibling With an ED

Interestingly, alongside the significant difficulties, non-ED siblings described some positive consequences of the disorder on both themselves and their family. In-depth interviews indicated that some siblings reported an increased sense of family solidarity and cohesion (31, 32, 37, 39), a closer relationship with the diagnosed sibling (31, 42, 43), and an improvement in family eating habits (42). In addition, many described the experience as contributing to the formation of a more resilient, mature, and responsible character, and as rendering them more empathic towards psychological difficulties (43, 47). In a phenomenological study, all 12 interviewees reported elevated sense of compassion and understanding towards others following their siblings’ disorder (31).

### Effects on the Family Environment

#### Family Dynamics

A common theme addressed by siblings in several qualitative studies was the dominance of EDs in family everyday life. While this theme does not refer directly to the non-ED sibling, but rather to the family as a whole, we did include it in the review as it is based on siblings’ reports. Thus, we believe it should be viewed as part of their experience as siblings, and as family members. Studies have shown that the ED is described by siblings as omnipresent, affecting every aspect of daily life and reigning family routine and conduct (25, 32, 43). Siblings often reported feeling family life revolved around the diagnosed sibling and the disorder, leaving little room for other issues, and creating a constant atmosphere of abnormalcy (28, 31, 43, 47). In interviews regarding family dynamics many said the disorder brought about a strained and volatile family climate, in which they had to constantly “walk on eggshells” (25, 28, 43). Siblings further described that the intense preoccupation with the diagnosed sibling’s mental and physical health often gave rise to arguments, notably between parents, as well as between the diagnosed sibling and the parents (25, 31, 32, 43, 47). As could be expected due to the nature of EDs, non-ED siblings saw mealtimes as the main arena for arguments and conflict, and some felt mealtimes dominated family life (28, 31, 32, 42).

#### Siblings’ Relationships With Family Members

##### Relationship With Diagnosed Siblings

A number of qualitative studies examined the effect EDs may have on the non-ED siblings’ relationships with each family member. First, most studies found non-ED siblings’ relationships with diagnosed siblings significantly changed due to the disorder (43, 46, 47). While some studies reported that non-ED siblings experienced stronger and closer relationships with their siblings (25, 28, 43, 46, 47), others described their relationships weakened, or increased in strain and tension (25, 28, 32, 46). Generally, non-ED siblings described an ambivalence towards their relationships with their siblings, which included both close and affectionate interactions, and conflictual and distancing communication (43). Nevertheless, one study found that when asked to quantitively rate their relationship with their sisters, non-ED sisters showed a negative correlation between positive and negative aspects of the relationship, suggesting a more split than ambivalent relation. Such correlation was not found for control participants, for whom the two scales did not exclude one another (40).

Qualitative studies found various factors straining this relationship. The most prevalent of those was difficulty in understanding diagnosed siblings’ feelings, thoughts, and behaviors (25, 32, 47), and at times viewing those as a manipulative and preconceived way of seeking attention (25, 32, 46). In several studies, non-ED siblings reported feeling neglected and underprivileged in comparison to their siblings, and expressed frustration over their experience of differential parental treatment (31, 43). However, it was also found that diagnosed siblings reported higher levels of jealousy towards their non-ED siblings than vice versa (36). In addition, some non-ED siblings described feeling uncomfortable around their siblings due to the disorder’s symptoms, especially the emaciated appearance, obsessive symptoms, and binge and purge cycles (31, 32, 47). Another reported difficulty had to do with non-ED siblings’ feeling that their eating habits and body were criticized by their siblings (32). Nevertheless, many also noted that their prominent feelings towards their diagnosed siblings were those of compassion, responsibility, and wish to protect (32, 42). Siblings’ relationships were improved when non-ED siblings had more knowledge of the disorder, and when they tended to see it as separate from the diagnosed sibling (39).

##### Relationship With Parents

As indicated by several qualitative studies reviewed, relationships between non-ED siblings and their parents were also often affected by the disorder. Again, while some non-ED siblings seemed to benefit from sharing their experiences and talking to their parents, and reported an improvement in their relationship (31, 32, 47), many described that the ED created an intricate and at times strained relationship. Non-ED siblings often felt parents were preoccupied with the diagnosed sibling and were thus less available, both emotionally and practically, leaving them feeling unnoticed and rejected (25, 39, 42, 43). In a study interviewing 12 siblings of chronic AN patients, non-ED siblings reported that they felt their diagnosed siblings received more parental attention and more practical and financial aid, both in everyday life and particularly during hospitalizations (31). Non-ED siblings also often reported that their parents demanded them to be independent and helpful at a young age, either in an indirect way or by explicitly asking them for consideration and compromise (25, 31, 43). These expectations, in turn, evoked at times feelings of anger and resentment (31, 43). Using semi-structured interviews, Dimitropoulos et al. (31) examined the ways parents handled the disorder and their effect on their relationship with non-ED siblings. The study showed that non-ED siblings described parental denial of the disorder as undermining their communication with their parents, leaving them feeling unacknowledged and their worries unvalidated. Participants also shared that parental accommodation of the disorder, such as allowing binges of food intended for the whole family, cleaning after purges, and preparing meals with nutritional restrictions, gave rise to feelings of parental inadequacy and anger towards them.

#### Siblings’ Family Role

The reviewed studies indicated that the changes in family relationships and everyday life brought about a change in non-ED siblings’ perceived roles in the family as well. As reported in several qualitative studies, non-ED siblings commonly assumed responsibility over their siblings’ health and well-being, especially when they were the older siblings (25, 32, 43, 46). Non-ED siblings described a dual and at times conflicting role, in which they were both supporters of the diagnosed siblings and the caring parents’ collaborators (25, 31, 32). They tried to balance protecting their siblings, collaborating with them, and keeping their secrets, with gathering information regarding their eating habits for their parents’ surveillance and care (25, 32, 43, 46), at times in response to parents’ direct requests (25, 31). Non-ED siblings reported serving as mediators between the diagnosed sibling and the parents, either when they felt close to both sides or when they felt their parents were ineffective in their coping with the disorder (31, 43). Another common role non-ED siblings assumed is that of support and care for the parents, particularly when parents were living apart (31, 42, 46). Non-ED siblings reported feeling worried for their parents’ well-being and feeling responsible for them (43). They also reported parents saw them as a source of help and knowledge and consulted them about the disorder (31). A third common role non-ED siblings assumed is that of “the healthy child,” emphasizing their achievements and coping, and hiding their difficulties (49). Non-ED siblings reported deliberately concealing their own needs, worries, and distress from their parents in an effort to avoid burdening them (25, 31, 32, 39). These efforts to conceal difficulties may be successful at times, as one study found parents’ report of non-ED siblings’ levels of emotional difficulties were similar to the norm, while the non-ED siblings’ self-report indicated higher levels of distress (44).

### Interaction With Health Professionals

In qualitative studies, siblings’ opinions regarding their own inclusion in the disorder’s management and treatment were mixed. Some siblings did not want to be included at all, yet many wished to be more involved and informed (42). Often siblings felt left out of the therapeutic process, even when they participated in family sessions (32, 43). In a study interviewing siblings after participation in family therapy, many siblings reported they initially attended family sessions, but their attendance reduced over time, partly because they felt they were not a part of the treatment process. Other common reasons for the decrease in attendance were boredom and preferring to participate in social interactions and other commitments (46). In another study, some siblings reported they did not understand why they were supposed to attend family sessions, and found the experience uncomfortable (47). However, in both studies siblings reported that, in retrospect, family therapy has been a beneficial experience for them. Most siblings wished they had attended more sessions, and found that therapy gave them hope and a better understanding of the situation (46, 47).

Another common finding was non-ED siblings’ need for individual counseling (31, 43). Many siblings also expressed a wish to meet with others in a similar situation to their own, and to attend siblings’ support groups (31, 42, 43)

### Coping Strategies

Non-ED siblings described a wide variety of ways of coping with the disorder’s ramifications. A recurring strategy in many qualitative studies was distancing oneself from the stress and conflict generated by the disorder (25, 31, 32, 47). Thus, non-ED siblings often attempted to maintain a normal everyday life despite the difficulties at home, taking “time-out” from the strained atmosphere (39, 42). Some turned to friends, neighbors, or romantic partners for support, and saw them as an oasis of normalcy (25, 31, 39, 42, 47). In one study, some participants said they took comfort in the anticipation of moving out and living independently in the future, a thought they found liberating (25). Another coping strategy that siblings found to be helpful was learning about EDs and their siblings’ experience (25, 39, 43, 47). A better understanding of EDs seemed to help siblings distinguish the disorder from the sibling him/herself, and improved their relationships with them (31, 39). Additionally, some siblings used rationalization, as they expressed understanding and acceptance for receiving less attention due to the situation (47).

### Factors Moderating the Effect of EDs on Siblings’ Well-Being

Only five of the reviewed studies examined possible moderating factors of the disorder’s impact on non-ED siblings, concentrating on several familial and situational factors. Non-ED siblings living with the diagnosed siblings were found to be more vulnerable to the detrimental effects of the disorder (39). In addition, in families characterized by lower levels of functioning and less social support, and in which parents were ineffective or in denial regarding the disorder, non-ED siblings were exposed to more negative emotions and reactions (31, 36). Illness duration was also found to be related to the non-ED siblings’ level of well-being (44). Female and older siblings were found more likely to take an active role in caregiving, and thus were more exposed to the disorder’s effects (43).

## Discussion

The aim of this paper was to review the existing literature concerning siblings of individuals suffering from EDs. To our knowledge, this is the first attempt at exploring and organizing the existing findings on this group. This effort comes at a time where researchers and clinicians alike are showing growing interest in the effects of psychopathology both on the family as a whole, and on non-diagnosed siblings specifically. Over the years, attention to family members of individuals suffering from psychopathology was mostly directed at parents and offspring. This oversight is surprising considering the importance of the sibling relationship to one’s development, and the risks siblings are exposed to as a result of their unique familial position. Siblings often experience the burdens of living with a family member suffering from psychopathology, and may strongly feel its effects on parental and family dynamics. Their lives are frequently greatly affected by the situation, and as sibling relationships are usually the longest relationships individuals keep throughout their lives, it is a struggle likely to accompany them for years to come. Thus, it is of great importance to gain further insight into siblings’ experiences and needs in context of EDs.

Studies on the effects of other psychopathologies on the family (e.g., 50) reveal consequences for individual family members, in terms of personal well-being and manifestations of psychopathological symptoms, as well as for the family system as a whole. As illustrated in this review, EDs similarly affect non-ED siblings, in various ways.

While there is wide agreement that patients’ EDs may have an effect on non-ED siblings, findings regarding the nature and magnitude of this effect remain mixed. Overall, it appears that many siblings’ quality of life is impaired due to the disorder, and difficult feelings such as fear, guilt, sadness, and anger were common in most studies. However, studies remain divided as to the effect of EDs on non-ED siblings’ level of psychopathology. As is the case with family studies in other disorders (e.g., PTSD; 16), findings on EDs and siblings may be divided into manifestations of specific ED-related symptoms, and generalized psychopathological symptoms and distress.

With regards to EDs symptoms, while some studies reported no differences between non-ED siblings and controls in body image and eating-related behaviors and beliefs, others indicated a higher inclination to sub-clinical levels of EDs, and an experience of difficulty around food among the former. This warrants further research as the former findings are not in line with existing literature, which indicates ED symptoms tend to spread among peer groups, in both normative and hospitalized populations (51, 52). In order to understand the moderating factors accounting for the differences found in non-ED siblings’ level of ED symptoms, there is need for research concerning the mechanisms underlying the spread of ED symptoms in these families. As this review indicates studies in this group are scant, yet the study of the spread of EDs among other peer groups has gained some attention. On the behavioral level, numerous studies found people tend to unwittingly mimic their companions’ food intake (53). This is a potential mechanism for the spread of EDs, particularly because such mimicry is more prevalent among women, and even more so because lean individuals are more likely to be mimicked (53). Additionally, exposure to weight loss-encouraging talk, which is frequent among individuals with EDs, can strengthen unhealthy food and body related cognitions. Such conversation patterns, termed “Fat Talks,” were found to be related to negative affect, depression, and feelings of guilt (54), as well as to ED-related behaviors and cognitions such as body dissatisfaction and bingeing and purging behavior (55).

Findings regarding psychopathologies other than EDs were also highly mixed, ranging from no reported differences between non-ED siblings and controls, to reports of higher levels of depression, obsessive behavior, and even suicide. Alongside the great burden of living with a family member suffering from psychopathology, non-ED siblings are repeatedly exposed to the variety of negative emotions and mental states experienced by their siblings. This exposure may put them at elevated risk for the development of various psychopathologies, since studies conclude that people in close relationships often come to share a similar baseline level of both positive and negative emotions and a similar mental health state (56).

Looking beyond psychopathological symptoms, studies show that family relationships and dynamics are also greatly affected when a family member suffers from an ED. These findings are in line with existing literature regarding siblings of individuals with other psychopathologies, indicating non-diagnosed individuals may feel their siblings’ psychopathologies cause stress in the family, disrupt household routines, and impact relationships (57). Non-diagnosed siblings in such families describe feelings of grief over the loss of the relationships they had with their siblings before the psychopathology onset (58), and experience similar degrees of grief as parents do (59). Changes in the relationships with parents are also common among siblings, and similarly to siblings in the reviewed studies, many report feeling that due to their parents’ preoccupation with the diagnosed sibling, they themselves receive less parental attention, and feel invisible, abandoned, or forgotten by their families (49, 60).

Studies in this review also found that non-ED siblings’ position and role in the family may change on account of the disorder. The common roles described in this review are similar to those reported by studies concerning other psychopathologies, including serving as “go-betweens,” or mediators, between diagnosed siblings and parents, and playing a supporting role for parents (49). In addition, non-ED siblings often carry the role of “the healthy child” in the family, frequently emphasizing their achievements, and concealing their struggles. Due to this combination, their difficulties could often be masked and neglected. The concealment and secrecy typical of EDs might strengthen non-ED siblings’ tendency to take on these roles, and aggravate the risk of overlooking their difficulties.

Interestingly, non-ED siblings also describe some positive consequences their siblings’ disorders have on them and their families. Some mention a contribution to their personal development and character, and a positive impact on family solidarity and cohesion. It should be noted that such effects, showing that adversity may in fact contribute to one’s personal growth, were also found in other areas of psychopathology (61). These salutogenic effects may prove to be important, and warrant further research.

Siblings coping strategies reported in the reviewed studies were usually in the realm of acquiring knowledge about the disorder, and accordingly many expressed their hope that professionals would provide more information on the subject. Another common strategy was distancing oneself from one’s home and siblings, thereby avoiding being in touch with the disorder and its implications. Both strategies were also found common and effective for siblings of those suffering from psychopathologies other than EDs (11).

### Methodological Limitations of Existing Studies

Our review also raises several methodological concerns in this area of research. First, there are no clear and consistent inclusion criteria of the study group across studies. Participants’ age and birth order were varied, and seldom controlled for, and the criteria according to which a specific sibling in families with multiple children was chosen for the study were rarely mentioned. Furthermore, even though gender is a notable moderating factor for EDs and for caregiving behaviors (62, 63), many studies included both male and female subjects without accounting for differences, thus limiting the examination of the effect of gender on EDs’ impact on siblings. In addition, no study has focused exclusively on male siblings—a topic that clearly warrants further research.

Another inconsistency across the reviewed studies concerns the ED type and characteristics. EDs are a varied family of disorders, and each might give rise to different challenges and consequences for non-ED siblings (64). The only ED diagnosis to receive specific attention was Anorexia Nervosa, while other studies included siblings of patients with various EDs. In these studies results were not analyzed separately for each ED, possibly due to small sample sizes. The lack of studies specifically examining EDs other than AN precludes comparison and insight into the unique impact of each ED on non-ED siblings. In addition, co-morbidities, which are highly common among ED patients (1, 63), were not controlled for in any study, thus preventing any clear conclusions as to the unique role of the EDs in siblings’ distress (as opposed to, for example, co-occurring depression or anxiety symptoms).

An additional aspect of EDs which was not accounted for is the disorders’ duration and current treatment status. Chronic conditions and newly diagnosed EDs have different expressions and different prognoses (65), possibly affecting the disorder’s impact on siblings. Additionally, most studies recruited participants whose siblings were hospitalized or were out-patients in ED clinics. Thus, there is a gap in knowledge regarding participants whose siblings refuse treatment, or rather are in a more stable state. Similarly, some studies obtained patients’ consent for the participation of their siblings, and were therefore exposed to selection bias based on the nature of the siblings’ relationships.

A serious methodological limitation in some of the reviewed studies lies in the omission of siblings with past or present ED diagnosis or symptoms. It is reasonable to assume that these studies excluded the most vulnerable group among siblings. This may impede identification of the factors distinguishing non-symptomatic, sub-clinical, and ED-diagnosed siblings, thereby limiting our understating of risk and resilience factors among siblings.

Another significant limitation of previous studies has to do with variable selection. As shown in this review, studied factors moderating EDs’ impact on siblings’ well-being and psychopathology were to date mostly situational (e.g., birth order, living status). To our knowledge, no study to date has examined the effect of intrapersonal variables on non-ED siblings’ resilience or risk to psychopathology. As evident from other psychopathologies, some intra- and interpersonal factors may render family members more vulnerable to these disorders’ effects. A notable aspect found to affect one’s susceptibility to the transmission of symptoms within the family are patterns associated with the regulation of interpersonal distance, such as attachment, family borders, and differentiation of self (e.g., 66). These variables may be especially relevant in families with a member suffering from an ED, as clinicians and researchers suggest that difficulties in separation and individuation often play a significant role in the etiology and maintenance of EDs (67). Indeed, several studies of clinical and non-clinical samples found low levels of differentiation were predictive of elevated ED symptomatology (68, 69)

### Theoretical and Clinical Implications

Siblings of individuals suffering from EDs are a scarcely studied, yet considerably at-risk group. To our knowledge, this is the first paper aimed at reviewing the existing literature on the subject, and it reveals the limited state of knowledge related to this issue. As with other psychopathologies and conditions, the study of siblings has only recently started developing and expanding. Only half of the studies reviewed in this paper pertain specifically to siblings and their experiences, while the rest compare them to controls as a means to expand knowledge regarding EDs. Research focusing on siblings of those with EDs can join and enrich the growing body of knowledge on the spread of psychopathological symptoms in families. Thus far, studies have concentrated mainly on spouses and offspring, and dealt with psychopathologies other than EDs, for example depression and PTSD (e.g., 15, 18). Examining siblings in the context of EDs in the family could help identify unique and shared characteristics or mechanisms of symptomatic spill-over in the family across different disorders and kinship.

This review clearly indicates that more studies are called for in order to broaden our understanding of non-ED siblings and their experiences. As the existing qualitative and quantitative findings reveal contradictory pictures at times, it appears there is need for research methodologies that could integrate them to create a more multilayered understanding. Mixed methods studies, of which there was only one in the reviewed studies, could prove a beneficial way to study the complexity and provide a more intricate description at both the symptomatic and subjective levels. Furthermore, future studies are encouraged to take into consideration the great heterogeneity in previous work, either by narrowing inclusion criteria, or by accounting for different moderating factors. Such variables could be related to the disorder itself, examining the effects of ED type and duration, treatment status, and co-morbidities. They could also pertain to characteristics of non-ED siblings themselves, such as gender and interpersonal relating patterns. In order to identify potentially significant factors, we encourage researchers to draw at least some knowledge from studies of other psychopathologies, which have looked at a wider array of psychological variables. Uncovering possible moderating factors for psychopathology in siblings of individuals with EDs may help in constructing a more comprehensive model of risk and resilience factors in this group. For such a model to be accurate and externally valid, future studies should also avoid exclusion of participants with past or present diagnoses of psychopathology.

This review also holds clinical significance. The literature covered in this paper indicates that siblings of patients with EDs may be seen as facing an increased risk of developing ED symptoms, and are a risk group in need of attention. This may be attributed to shared genes and environment—factors which were beyond the scope of this paper, but also to intense siblings’ continuous exposure to EDs, which may be somewhat “contagious.” Understanding non-ED siblings’ experience and needs can aid in early detection of psychopathology, and in the identification of those siblings who are particularly at risk of developing EDs, which is crucial as early diagnosis and intervention potentially lead to a more positive prognosis (70). In addition, as qualitative studies indicate, siblings of persons with EDs tend to avoid disclosing distress to their parents or seek professional help. Hence, mental health professionals are encouraged to more effectively and proactively identify the needs and challenges faced by this vulnerable population.

Furthermore, it appears siblings of ED patients receive little clinical attention. Despite family therapy being a common and recommended treatment for EDs (5), to our knowledge little has been written about non-ED siblings’ involvement and experience in it (46, 47). Most references to non-ED siblings in this context have to do with their contribution to their siblings’ recovery, thus failing to address the needs of the non-ED sibling (71). The relative silence of clinicians and researchers around this subject stands in contrast to non-ED siblings’ common wish for more professional help. Therefore, it would be beneficial for professionals to more closely monitor siblings’ well-being and reach out to offer support and personal therapy as needed. Since, as mentioned, common treatments of EDs are family-oriented, family therapists are likely to be in a position to play this role. Moreover, a more accurate recognition of siblings’ needs could be translated into helpful psychoeducation for parents and into a better way to engage siblings in therapy. In addition, interventions specially designed for non-ED siblings have been almost entirely absent in empirical and clinical literature. Better insight into non-ED siblings’ experiences could contribute to the development of specialized interventions for this group.

### Conclusion

This review leads to conclusions on theoretical, methodological, and clinical levels. It can be concluded that expansion of the scarce knowledge existing regarding non-ED siblings is necessary. As has been done with other psychopathologies, better understanding of non-diagnosed siblings’ experience and psychopathology can shed light on pathological family dynamics, and the phenomena of symptoms spill-over in the family. Methodologically, such studies should be more meticulously designed, possibly including both quantitative and qualitative data. As can be surmised from the present review, non-ED siblings are also in need of more clinical attention. We encourage clinicians in both family and personal therapy settings to be aware of the possible risk factors of this group, in order to identify distress at an early stage and intervene accordingly.

## Data Availability Statement

The original contributions presented in the study are included in the article/supplementary material. Further inquiries can be directed to the corresponding author.

## Author Contributions

All authors took part in conceptualizing the ideas in the review. IM wrote the first draft of the paper and conducted the literature review. DH and YG contributed in editing and revising the manuscript.

## Conflict of Interest

The authors declare that the research was conducted in the absence of any commercial or financial relationships that could be construed as a potential conflict of interest.
